# Case report: Avoidant/restrictive food intake disorder after tonsillectomy

**DOI:** 10.3389/fpsyt.2024.1351056

**Published:** 2024-06-27

**Authors:** Gellan K. Ahmed, Ahmed A. Karim, Eman M. Khedr, Khaled Elbeh, Amira Moheb, Marwa Abokresha, Nourelhoda A. Haridy

**Affiliations:** ^1^ Department of Neurology and Psychiatry, Faculty of Medicine, Assiut University, Assiut, Egypt; ^2^ Department of Child and Adolescent Psychiatry, Institute of Psychiatry, Psychology and Neuroscience, King’s College London, London, United Kingdom; ^3^ Department of Psychiatry and Psychotherapy, University Hospital Tübingen, Tübingen, Germany; ^4^ Department of Internal Medicine, Gastroenterology Unit, Faculty of Medicine, Assiut University, Assiut, Egypt; ^5^ Department of Neuromuscular Disorders, University College London (UCL) Institute of Neurology, London, United Kingdom

**Keywords:** avoidant/restrictive food intake disorder, tonsillectomy, child and adolescent psychiatry and psychotherapy, cognitive-behavioural therapy, psychopathology, children

## Abstract

**Background:**

Avoidant Restrictive Food Intake Disorder (ARFID) is a newly classified eating disorder that requires further understanding of its presentation. There is no previous report of ARFID in a child post-tonsillectomy. ARFID may be a potential negative outcome for children following oropharyngeal surgery.

**Case presentation:**

A female child aged 10 years and 2 months presented with ARFID associated with depression, anxiety and nutritional deficiency following tonsillectomy. She had more difficulty in swallowing solids than fluids and had repeated vomiting and spitting food after chewing it. She became dehydrated and malnourished with a BMI of 10.5 and was misdiagnosed with myasthenic gravis.

**Conclusions:**

To our knowledge, this is the first case report of ARFID in a child post-tonsillectomy. We discuss the pathophysiology of ARFID, which remains elusive, and recommend psychiatric assessment when evaluating children post operative tonsillectomy.

## Background

Avoidant/restrictive food intake disorder (ARFID) is characterised by the avoidance or restriction of food ingestion in terms of quantity and/or variety. This can lead to weight loss or stunted growth, nutritional deficiencies, reliance on enteral or supplemental feeding (i.e., feeding through a tube or oral supplementation that is not medically required or provides at least 50 percent of daily caloric intake), and/or impairment of psychosocial functioning (1). The DSM-5-TR offers three examples of ARFID profiles: (a) sensory sensitivity, (b) fear of negative consequences, and (c) lack of interest in food/eating (2).

Here, we present a child who presented with ARFID associated with depression and nutrient deficiency following tonsillectomy. She exhibited food avoidance or restriction due to a fear of aversive consequences, such as a fear of choking, vomiting, or gastrointestinal pain. Often such individuals have experienced a food-related trauma and subsequently begin avoiding the index food to guard against another negative experience. While the avoidance reduces anxiety momentarily, it reinforces anxiety over time by preventing the opportunity for new corrective learning to occur (3).

The possible connection between fear of eating or avoidance of eating after a tonsillectomy may be attributed to a variety of factors, including the following. Discomfort and pain can significantly impede the efficacy of swallowing and eating. Glossopharyngeal neuralgia, although rare, can cause severe pain (4). Despite the significant discomfort children often feel post-tonsillectomy, there is persistent evidence of inadequate pain management (5). This may result in a fear or avoidance of eating to prevent additional discomfort or agony. Also, bleeding risk may increase when consuming solid foods or eating shortly after surgery (6). This concern of inducing bleeding can lead to a hesitation to consume food.

Moreover, dehydration and nausea frequently occur following a tonsillectomy (7), causing a lack of desire or even aversion to eating, which can result in a dread of eating. Negative experiences and recalling paediatric pain can have lasting effects. Children who remember pain with a larger negative bias, where the recalled pain is greater than the initial pain report, have worse pain outcomes (8). This challenging or traumatic experience during or after a tonsillectomy, may cause dread or anxiety related to eating, and potentially triggering memories of the unfavourable event. Furthermore, preoperative caregiver anxiety is strongly correlated with postoperative pain in children receiving elective, ambulatory surgery in low- and middle-income countries (LMIC). Efforts to decrease caregiver concern should be a crucial aspect of the comprehensive therapy of postoperative pain in children (9).

The current case report aimed to demonstrate the association between ARFID and a particular oropharyngeal operation, tonsillectomy in children. We also speculate on a potential pathophysiological link between the two conditions.

## Case presentation

A 10-year-old girl was brought to the psychiatry outpatient clinic at Assiut University in Egypt by her mother as referred by her paediatric doctor. The patient was a fourth-grade primary school student. She is the fourth of six children in the family. She appeared well-dressed but underweight, with long hair in the frontal part of her scalp. Examination revealed dry tongue, swollen parotid glands, hostile looks towards her mother, and a depressed mood and affect. Her body mass index (BMI) was 10.5. According to the mother, a 35-year-old housewife with a secondary education, the patient’s physical and mental development were normal, and she excelled in her schoolwork. However, on August 23^rd^, 2022, four months prior to the current presentation, the patient underwent a tonsillectomy operation. She claimed to have been aware of everything during the operation instead of receiving general anaesthesia, although she did not experience any flashbacks, nightmares, or distressing external reminders. The patient was discharged home on the same day of the tonsillectomy. At discharge, doctors prescribed intravenous antibiotics, nonsteroidal anti-inflammatory medications, and analgesics.

In the first two days following the tonsillectomy, the patient complained of difficulty swallowing and stopped eating and drinking, refused to speak or make any sounds, and spent most of the day sleeping. On the third day, she began drinking fluids and speaking but was unable to eat solid food. The mother sought a paediatric consultation, and the doctor gave the patient intravenous fluid and advised the parents to consult an ear and nose and thorax (ENT) doctor. On the fifth day following the operation, the mother sought an ENT consultation, who diagnosed the child with postoperative inflammation characterized by a whitish discoloration at the site of the operation. The doctor also advised the parents to force the child to consume and to continue the treatment with antibiotics. Parents occasionally attempted to motivate her and occasionally coerced her to consume. However, over the next few weeks, the patient continued refusing to eat and repeatedly vomited despite being hungry, claiming sore throat pain.

On September 25, 2022, the girl began consuming small amounts of semisolid food (2 teaspoons per day), but experienced repeated vomiting, and lost around 5 kilograms weight in one month. Her weight dropped to 13 BMI despite receiving IV fluids. In October 2022, upon returning to school, the patient exhibited a sad mood and cried most of the day after returning home, as her friends commented on her body and bullied her. She refused to attend school or study due to fatigue and weakness. She stopped playing with her toys which were her favourites before, preferring to watch television or YouTube channels for children without paying attention to the content. After consulting with several ENT specialists, a laryngoscopy was performed, and no abnormalities were identified. During the subsequent appointment to the ENT specialist, the girl was compelled to ingest and swallow food while in the doctor’s presence. Despite experiencing discomfort from swallowing without vomiting, the doctor informed the parents that the patient’s issue had been resolved. Despite facing numerous challenges, the patient continued to refuse food, experienced recurrent vomiting at home, and her condition worsened, resulting in a further weight loss of 4 kg over three weeks.

**Figure 1:**
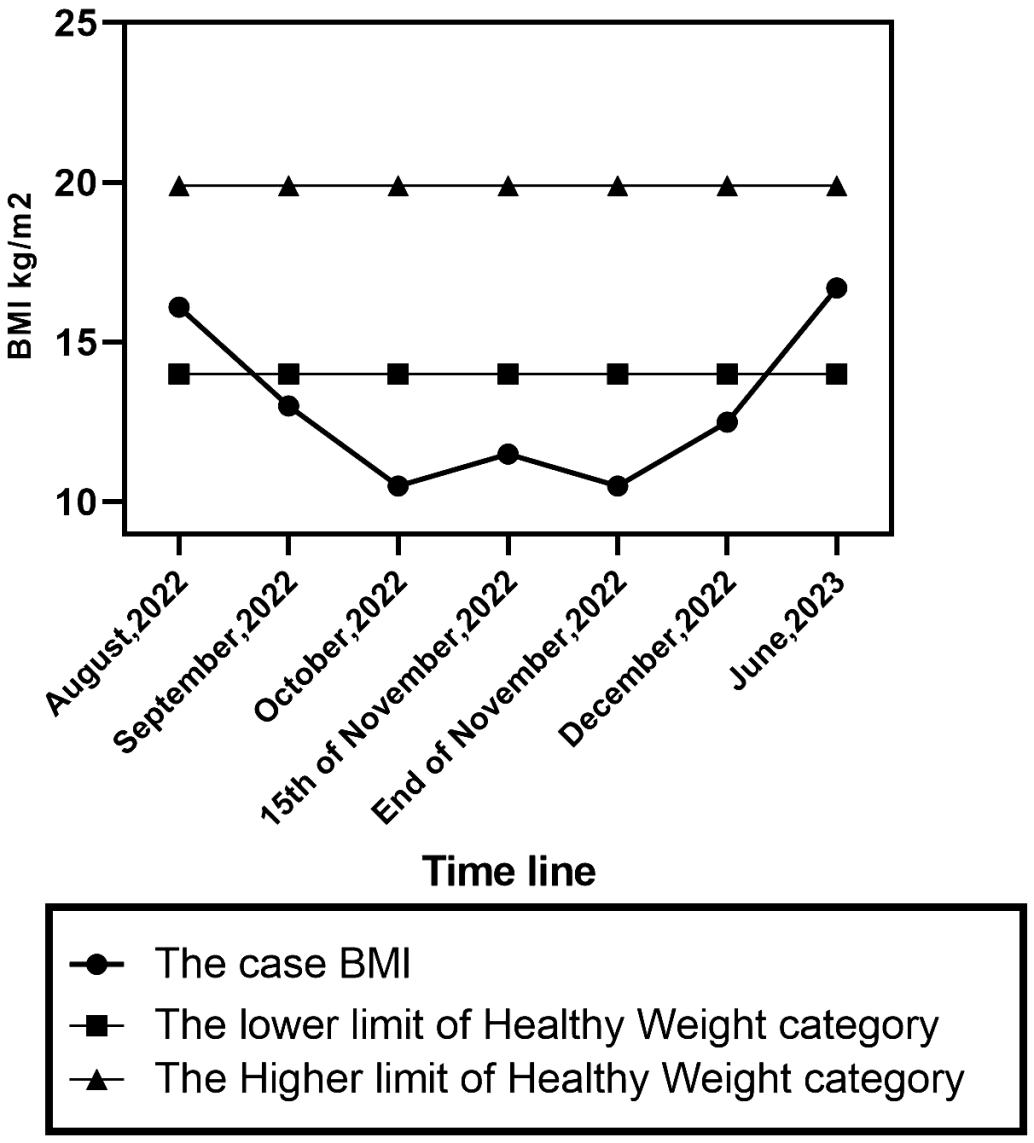
BMI percentile changes of the case.

On October 15^th^, 2022, she was admitted to the paediatric department at Assiut University Hospital for one month, receiving intravenous fluids and Ryle feeding. During her hospital stay, the patient imitated another child’s behaviour of chewing food and spitting it out. She refused to let her mother eat in front of her and asked her to fast. She did not experience any concerns related to body image, weight gain, or the sensation of a lump in the throat. She was observed to have no food avoidance behaviour; rather, she incessantly requested food to be placed next to her so she could chew it before spitting it. A multidisciplinary team, including neurologists, internal medicine specialists, ENT doctors, and paediatricians, was consulted. Extensive laboratory, imaging, and neurophysiological studies, including repetitive nerve stimulation test (RNS) to exclude myasthenia gravis, were performed. RNS test showed positive results in one muscle and negative in another, leading to a diagnosis of myasthenia gravis. At discharge, she was prescribed pyridostigmine 30 mg every 6 hours for 2 weeks. The patient’s weight was 11.5 BMI (initially 10.5 BMI upon admission) (**Figure 1**). Two weeks later, there was no improvement in her symptoms and her paediatrician referred the patient to the neuropsychiatry department at Assiut University for further evaluation and management.

### Physical examination and investigations

While she was hospitalised, her vital signs were normal. She reported constipation, abdominal cramp, acid reflux, dry skin, dry and brittle nail, and thinning hair on head. Her BMI was (10.5).

Laboratory investigations were performed, including serum electrolytes (Ca, MG, Na, and K), complete blood count, renal, liver function, and measuring serum Acetylcholine receptor antibodies level all were in the normal range except iron deficiency anaemia.

Electrocardiogram (ECG) was normal and decrement test for Myasthenia Gravis (repetitive nerve stimulation) was positive in frontalis muscle and negative in biceps and triceps.

Regarding imaging studies, the patient had a barium swallow, barium meal, and gastroscopy with no abnormalities. Also, cervical MRI was done but no abnormalities were detected apart from bilateral cervical lymphadenopathy (largest size:0.5 cm).

The total score of Wechsler Intelligence Scale for Children-3^rd^ edition (WISC) was 82. Revised Children’s Anxiety and Depression Scale (RCADS)-parent version was performed and identified clinically significant for anxiety and depression. She was assessed using the parent version of the Eating Disturbances in Youth Questionnaire (P-EDY-Q) and the Parent Eating Disorder Examination Questionnaire (PEDE Q) (10, 11). The P-EDY-Q (by maternal responses) was consistent with ARFID for avoidance of foods due to items 4,6,7,10.

According to DSM- 5 the girl was diagnosed by the interprofessional psychiatry team as (first diagnosis): avoidant restrictive food intake disorder (ARFID and major depressive disorder, single episode, severe with need for further assessment (12).

### Treatment

A Ryle tube insertion for feeding was inserted. The patient was prescribed a daily dose of 25 mg of oral SSRI (Fluvoxamine). After four days, the dosage was increased to 50 mg, as recommended for children and adolescents. Parent psychoeducation and behavioural parental training were scheduled. Exposure therapy for the patient was arranged. The patient was advised to engage in slow breathing and muscle relaxation exercises. The patient was advised to progressively increase the size of each mouthful and decrease the amount of chewing movements per bite. Cognitive behavioural psychotherapy (CBT) sessions were linked with exposure therapy. Treatment resulted in increased oral intake, decreased mealtime, and less fear.

### Outcome and follow up

One month following the patient’s admission to the psychiatry unit. She was discharged with a BMI of 12.4 and achieved full recovery over six weeks of follow-up CBT psychotherapy. The diagnoses at the time of discharge were confirmed the first diagnosis. Six months later, her BMI was 16.7.

## Discussion and conclusions

Our preliminary diagnosis for this case study is ARFID with fear of aversive consequences and associated with depression, anxiety, and nutritional deficiency according to the DSM-5 TR (1, 2). A possible psychopathology is that vomiting in infancy due to infections or overeating can cause temporary fear (13). In addition, forcing children to eat exacerbates the symptoms. Infants adopt avoidance behaviours and refuse to eat when afraid, leading to a vicious cycle of anxiety, gastrointestinal symptoms, and body-related anxiety. Also, preexisting family conflicts and the subsequent family response significantly influence the disorder’s severity. To better comprehend the psychopathology of ARFID, it is necessary to divide it into two categories: post-traumatic type with an acute onset and phobia-related central symptoms, and gain-illness type with triggers but core symptoms involving conversion and necessitating environmental manipulation to alleviate symptoms (13).

In the current case, several challenging factors were present: Firstly, the patient complained of throat pain and difficulty swallowing solids more than fluids, initially directing medical attention toward physical issues. It is essential to identify carefully any inconsistencies in describing a patient’s fear, especially when the patient has difficulty communicating, as children often struggle to articulate their specific fear (14). Secondly, the patient exhibited an obsessive temperament, which might increase vulnerability to developing ARFID. Individuals with obsessive premorbid personalities tend to be treatment-resistant (13).

Thirdly, the onset of food refusal following a traumatic incident is not clearly defined and might be linked to the severity of anxiety or other unknown conditions. The time between the onset of symptoms and seeking consultation can range from five weeks to thirty years, and it is uncommon for patients to have received treatment prior to seeking assistance (13, 14). The intensity of ARFID symptoms and the types of food allowed vary with fear. Environmental pressures and events in life, such as professional, educational, or familial stress or physical illness may also cause or worsen the fear. Chronic subthreshold presentations, frequently unrelated to a specific traumatic incident, are characterized by a more slow and progressive illness that may include multiple foods (13, 14).

Furthermore, preoperative caregiver anxiety is strongly correlated with postoperative pain in children, as reported in a previous cross-sectional, descriptive study for children aged 4–12 years undergoing elective ambulatory tonsillectomy or adenotonsillectomy in a low- and middle-income countries (LMIC) (9). Efforts to decrease caregiver concern should be a crucial aspect of the comprehensive therapy of postoperative pain in children (9).

### Medical evaluation

Medical evaluation is a very important step in assessment of cases with ARFID (3). ARFID causes dietary deficits, electrolyte imbalances, bradycardia, gastrointestinal issues, amenorrhea, and bone loss (15). Anthropometric measurements with setting of weight targets for underweight patients requires assessing growth history and requirements for weight increase and/or height growth, especially for children and adolescents (15). Nutritional deficiencies should be examined since untreated nutritional deficits in ARFID can cause various problems, such as multivitamins, zinc, iron, or protein deficits. Self-report dietary recall or blood investigations can assess deficits (16). Evaluation also involves assessing the patient’s medications and other medical issues that may affect feeding. Food allergy evaluation is vital, especially for those with a fear of unpleasant outcomes such as an allergic reaction. Food allergy testing determines if the feared food can be reintroduced in-session. Other medical comorbidities include oral-motor and gastrointestinal disorders including celiac and Crohn’s disease should be evaluated (15).

A recent study contributes to the understanding of the clinical manifestations of ARFID patients and may facilitate the development of assessments (17). Although the current case underwent a comprehensive evaluation, certain investigations recommended in this previous study (17) were not executed, such as the bone density assessment, due to its limited availability at institutions with lengthy waiting lists.

### Differential diagnosis of ARFID

*Anorexia Nervosa (AN):* ARFID patients fear eating, while AN patients fear the consequences of eating, such as weight gain and body shape changes (13).

*Functional Dysphagia (FD):* Characterized by digestive difficulties, particularly with solids, and some patients may experience severe illness (13). Individuals suffering from hypersensitive nauseous reflex or globus hystericus may also experience difficulty swallowing without concern of choking (18). The diagnostic criteria include inability to swallow water or solids for more than two weeks, with no evident organic changes to the gastrointestinal tract, and where psychosocial factors are believed to be contributing (13).

*Concerns Regarding Body Image in ARFID:* Individuals with ARFID are not immune to societal influences regarding body shape and weight (19, 20). Some studies (21, 22), have reported behaviours typical of other eating disorders, such as binge eating, self-induced regurgitation, and excessive exercise in ARFID patients (23). Clinical judgment is required to monitor and document any changes in the clinical presentation of ARFID (15).

Certain factors as food scarcity, cultural practices like religious fasting, food refusal resulting from organic disorders or other psychiatric disorders were excluded. To rule out the presence of organic disorders such as achalasia, diffuse oesophageal contraction, gastric ulcers, and gastroesophageal reflux, endoscopic evaluations were performed (24).

Associated symptoms in cases with ARFID should be explored. Patients with ARFID may demonstrate medical (oesophageal irritation, hyperactive gag reflex), psychosocial (dysfunctional family mealtime culture), and topographical (head posture, muscle tension, etc.) alterations in their feeding response (14, 25). Accompanying gastrointestinal symptoms like nausea, heartburn, and abdominal bloating may be present in children, along with reactive behaviours like screaming, angry outbursts, or lengthy silences (13). ARFID may be associated with generalized anxiety disorder, social anxiety, social dysfunction, or withdrawal. High comorbidity with other anxiety disorders, such as panic disorder related to swallowing, obsessive conditions, and separation anxiety, may also be present. Patients with ARFID frequently exhibit other symptoms, including depressive symptoms, emotional and behavioural consequences, distorted body image, and sleep disorders (14).

Misdiagnosis in cases of ARFID should be prevented to minimize delays in diagnosis and subsequent nutritional consequences. The current case was initially misdiagnosed as myasthenia gravis (MG) after ruling out all local causes of dysphagia through procedures such as laryngoscopy. Investigation for MG was conducted due to its inclusion in the list of potential causes for unexplained dysphagia. Following a borderline result of repetitive nerve stimulation (RNS), antibody testing was performed based on the positive RNS result. Upon reassessment and consultation with neuropsychiatry staff, a thorough evaluation revealed underlying psychiatric issues, leading to the consideration of ARFID. So, it is imperative for paediatricians to possess knowledge regarding the diagnostic criteria for ARFID, as well as the potential necessity of medical intervention and referral for psychological treatment for these patients (3).

Repetitive nerve stimulation (RNS) is a conventional electrophysiological method for diagnosing MG. Studies demonstrated that a 10% decrease in amplitude from the first to the fourth or fifth intravolley waveform while stimulating at 2 to 5 Hz is sufficient to diagnose MG (26). RNS sensitivity is 40–50% for generalised myasthenia gravis and 10–20% for oculobulbar illness (27–29). False positive RNS may be the result of several factors including technical errors especially with inexperienced hands (30), excessive gel may cause the stimulator to slip away from the nerve, resulting in false-positive results (31). In addition, it could be a result in disorders with secondary defect of neuromuscular junction transmission (eg, ALS) (30). Also, muscular fatigue cause positive response in RNS (32).

Treatment of cases with ARFID: Currently, there is no evidence-based treatment for ARFID due to its rarity. Cognitive-behavioural therapy (CBT) can be combined with an SSRI, such as fluvoxamine in the present case, and initially with benzodiazepines if anxiety is present (33). Involving the family in an integrated treatment plan and psychoeducation is considered essential for therapeutic success, particularly with children and adolescents (34). A recent review examined the existing research on psychological interventions for ARFID and the criteria used to assess the outcome improvement (35). The treatment options provided to the current case which included CBT, and family therapy interventions followed the proposed approach described in this previous review (35).

## Conclusion

Since its discovery, AFRD has received insufficient attention in research. The presence of clinical symptoms and phobic experiences that trigger it can pose challenges in diagnosis. Furthermore, the current psychiatric diagnostic classifications may not adequately recognize the disorder due to issues in nosology. The clinical overlap of various eating disorders characterised by food abstinence frequently leads to misdiagnosis., treatment failure, prolonged illness, and severe physical and psychological consequences. Timely identification of AFRD is crucial in preventing these serious outcomes. Additionally, it is important to consider a conceptual framework that acknowledges both acute and subthreshold presentations of the disorder, as well as its co-occurrence with other anxiety disorders such as panic disorder or social anxiety. While several behaviour therapy approaches have shown promise in achieving complete remission, further research is necessary to determine the most effective method and establish appropriate follow-up protocols. A multidisciplinary teams involving general practice, gastroenterology, paediatrician, ENT and psychiatry is fundamental (24).

The case highlights the importance of a comprehensive evaluation and multidisciplinary approach in diagnosing complex cases, particularly when initial assessments yield inconclusive or borderline results. The eventual diagnosis of ARFID underscores the need to consider psychological and psychiatric factors, especially in cases with unexplained food refusal. Paediatricians should understand ARFID diagnostic criteria and the probable need for medical intervention and psychological treatment for these patients (3).

## Data availability statement

The original contributions presented in the study are included in the article/supplementary material. Further inquiries can be directed to the corresponding authors.

## Ethics statement

Written informed consent was obtained from the minor(s)’ legal guardian/next of kin for the publication of any potentially identifiable images or data included in this article.

## Author contributions

GA: Writing – original draft, Methodology, Investigation, Formal analysis, Data curation, Conceptualization. AK: Writing – review & editing, Supervision, Conceptualization. EK: Writing – review & editing, Writing – original draft, Resources, Methodology, Investigation, Formal analysis, Data curation, Conceptualization. KE: Writing – review & editing, Methodology, Investigation. AM: Writing – original draft, Methodology, Investigation. MA: Writing – original draft, Methodology, Investigation, Data curation. NH: Writing – original draft, Methodology, Investigation, Data curation.
